# OX40, PD-1 and CTLA-4 are selectively expressed on tumor-infiltrating T cells in head and neck cancer

**DOI:** 10.1038/cti.2016.16

**Published:** 2016-04-15

**Authors:** Ryan Montler, R Bryan Bell, Colin Thalhofer, Rom Leidner, Zipei Feng, Bernard A Fox, Allen C Cheng, Tuan G Bui, Christopher Tucker, Helena Hoen, Andrew Weinberg

**Affiliations:** 1 AgonOx, Inc., Portland, OR, USA; 2 Earle A Chiles Research Institute in the Providence Cancer Center, Portland, OR, USA; 3 Providence Oral, Head and Neck Cancer Program and Clinic, Providence Cancer Center, Providence Portland Medical Center, Portland, OR, USA; 4 Department of Cell, Developmental and Cancer Biology, Oregon Health and Science University, Portland, OR, USA

## Abstract

The tumor microenvironment of squamous cell carcinoma of the head and neck (SCCHN) has been shown to be immune suppressive. Therefore, strategies aimed at overcoming this issue could have a positive therapeutic impact. Hence, we investigated the expression of the known immune-modulatory proteins OX40, programmed cell death protein 1 (PD-1) and cytotoxic T-lymphocyte-associated protein 4 (CTLA-4) in SCCHN on different T-cell subsets of tumor-infiltrating lymphocytes (TIL) to ascertain whether these proteins could potentially be targeted alone or in combination for future clinical trials. T cells from peripheral blood (PBL) and tumor were analyzed for the expression of OX40, PD-1 and CTLA-4 in 29 patients undergoing surgery. These proteins were all expressed significantly higher in T-cell subsets isolated from tumors compared with PBL of the same patient. OX40 expression was significantly greater in the TIL regulatory T-cell (Treg) population relative to conventional CD4 and CD8 TIL or the Treg isolated from PBL. PD-1 expression was increased in all T-cell subsets relative to PBL. CTLA-4 was also increased in all TIL subsets relative to blood, and similar to OX40, its highest level of expression was observed in the Treg TIL. The highest frequency of PD-1, CTLA-4 and OX40 triple-positive cells were found in the Treg population isolated from the tumor. We analyzed both human papilloma virus-positive and -negative patients and found similar levels and expression patterns of these two patient populations for all three proteins. These data suggest that there may be therapeutic advantages of targeting these pathways independently or in combination for patients with this disease.

The immune paradigm is to destroy harmful entities within the body (for example, pathogens and cancer); however, failure to do so can be deleterious to the host ultimately leading to chronic disease and/or death. Hence, understanding how pathogens and cancer exist within a lympho-replete host may help to define pathways useful in eliminating harmful entities and restoring the host to a disease-free state. In this study, we examined the immune system in patients with progressively growing head and neck cancer and we compared T cells isolated from the tumor vs the peripheral blood (PBL) for the expression of co-stimulatory and co-inhibitory receptors, OX40, programmed cell death protein 1 (PD-1) and cytotoxic T-lymphocyte-associated protein 4 (CTLA-4). We identified unique patterns of expression of these immune-modulatory receptors on T cells within the tumor of head and neck patients.

Antibodies (Abs) that target T-cell surface proteins have recently been shown to be effective agents with which to stimulate an antitumor immune response in preclinical and clinical settings.^1, 2, 3, 4, 5^ Checkpoint inhibitors, such as anti-CTLA-4 and anti-PD-1, block negative signals to the T cells causing enhanced proliferation of effector and memory T cells and increasing immune-mediated tumor lysis.^6^ A positive phase III clinical trial of anti-CTLA-4, which demonstrated enhanced survival in patients with metastatic melanoma, led to the recent Food and Drug Administration approval of this Ab and has renewed enthusiasm for immunotherapeutic approaches in cancer patients.^7^ Abs directed to PD-1 or PD-1 ligand has also produced complete and partial responses as well as durable stable disease in patients with cancer and has recently been approved in melanoma and lung cancer.^8, 9^ In addition to immune checkpoint inhibitors, Abs have also been developed that enhance T-cell function by increasing co-stimulation. Agonists to tumor necrosis factor receptor family members such as anti-4-1BB have shown clinical activity in early clinical trials, and there is ample preclinical evidence that T-cell co-stimulation via 4-1BB can induce immune-mediated rejection of tumors.^10, 11^ All of these agents are currently in clinical trials for a number of malignancies and have led to immunotherapy being dubbed the ‘cancer breakthrough of the year' in 2013.^12^

Anti-OX40 is another promising Ab that is currently in early-phase clinical trials for the treatment of cancer. OX40 is a member of the tumor necrosis factor receptor family and a potent co-stimulatory pathway that when triggered can enhance T-cell proliferation, memory and antitumor activity.^13, 14, 15^ It has been shown that the immune-stimulating properties of OX40 agonists can overcome some of the immunosuppressive properties within the tumor microenvironment (TME). OX40 agonists increase T-cell infiltration into tumors and decrease the proportion of suppressive macrophages, suggesting that anti-OX40 improves immune responses in tumor-bearing hosts.^16^ Injection of OX40 agonists leads to therapeutic responses in tumor-inoculated hosts in several preclinical mouse cancer models, including 4T-1 breast cancer, B16 melanoma, Lewis lung carcinoma and several chemically induced sarcomas.^16, 17, 18^ Recently, our group completed a first-in-human phase I trial of an agonist Ab to OX40, which was well tolerated, enhanced both humoral and cellular immunity and exhibited signs of antitumor activity.^19^

Patients with squamous cell carcinoma of the head and neck (SCCHN) have poor prognosis, which has not significantly improved in the past four decades. In addition to smoking and alcohol, human papilloma virus (HPV) infection is an important oncogenic risk factor. It is now known that HPV-unrelated SCCHN is a distinct biological and clinical entity, which responds much less favorably than HPV-related carcinomas to conventional therapies.^20^ We hypothesize that the immune-suppressed TME that characterizes SCCHN can be overcome by strategies that increase antitumor immunity through enhancing T-cell responses and may be an ideal disease where immune modulation will have a significant therapeutic impact.

In order to develop a scientifically sound strategy to increase T-cell effector function, we performed this investigation to provide a better understanding of the expression of OX40, PD-1 and CTLA-4 in progressively growing head and neck tumor-infiltrating lymphocytes (TIL) and PBL. Additionally, we evaluated whether immune-specific phenotypic differences existed in patients with HPV-positive vs -negative status. Using paired autologous TIL and PBL lymphocyte specimens, harvested from a series of patients undergoing surgery for SCCHN, we observed high levels of expression of OX40, PD-1 and CTLA-4 in TIL T-cell subsets. This expression was particularly high among the regulatory T-cell (Treg) TIL population in both HPV-positive and -negative patients. To our knowledge, this is the first report of OX40, PD-1 and CTLA-4 expression levels in the TIL and peripheral lymphocytes from SCCHN patients and provides rationale for combination therapy with these agents in this disease.

## Results

**Figure 1:**
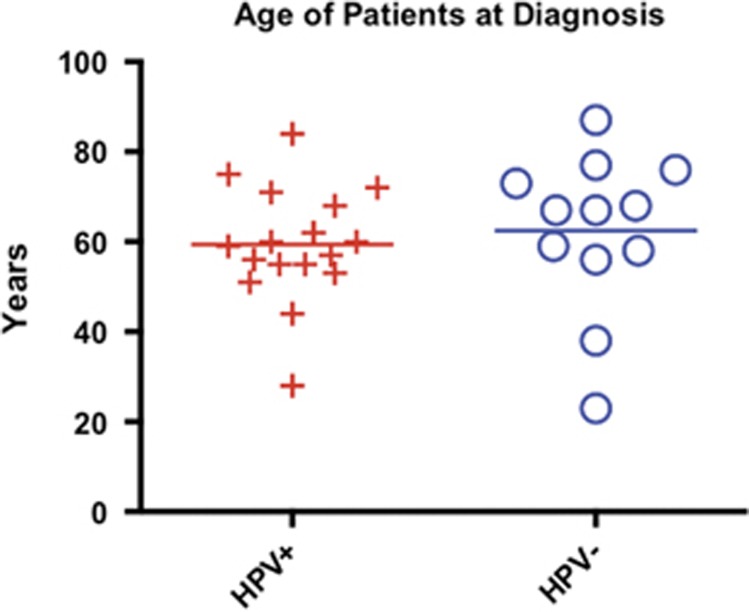
Age at diagnosis. Patients were stratified by HPV status and assessed for age at the time of diagnosis just prior to surgery. There was no significant age difference between the two groups of patients.

**Figure 2:**
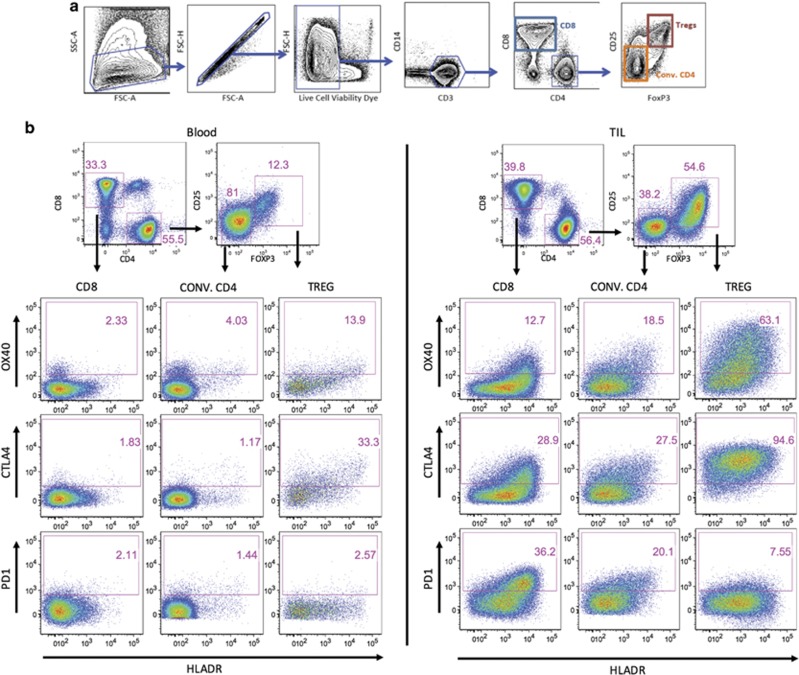
(**a**) Flow cytometry gating strategy: lymphocytes were obtained from the tumor and PBL of head and neck cancer patients and were analyzed by flow cytometry. A typical lymphocyte forward and side scatter gate was used to enrich for lymphocytes and exclude larger cells, such as tumor cells. A singlet gate was used to remove doublets from the analysis. Live cells are then selected by gating on the population that excludes the viability dye. Cells were then gated on CD3^+^ CD14^−^ cells to establish a pure T-cell population. This is then followed by the CD4 and CD8 gate. Tregs were identified by CD25-positive FoxP3-positive staining. This example was stained using TIL. (**b**) Tumor vs PBL: CD3 T cells were subfractionated into CD4 and CD8 T cells and the CD8, Tregs and conventional CD4s were assessed for OX40, CTLA-4 and PD-1 in the blood and TIL of cancer patients. Tregs were differentiated from the conventional CD4s using the CD25 and FoxP3 markers as shown above. Expression of OX40, CTLA-4 and PD-1 were assessed in combination with the activation marker HLA-DR in the three T-cell subsets. It should be noted that, in the subsequent histogram figures and throughout the rest of the manuscript, we use this T-cell gating strategy.

**Figure 3:**
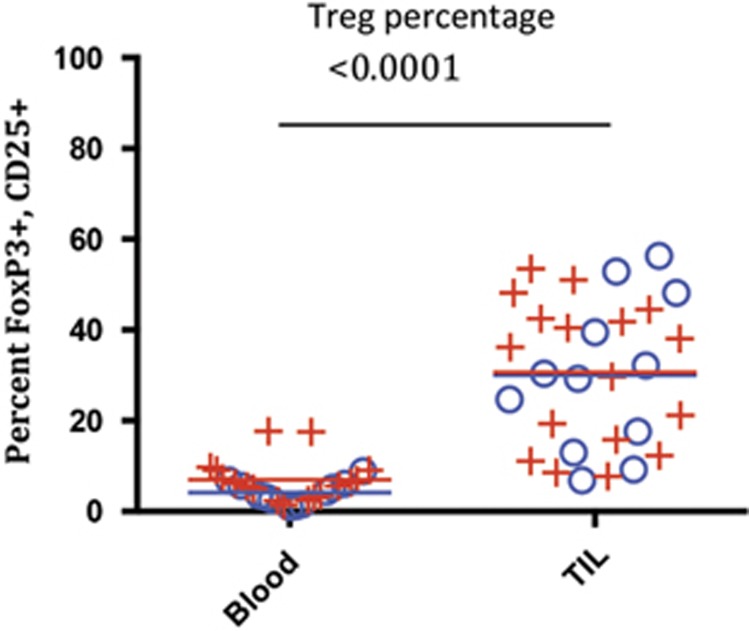
Treg percentage of total CD4 cells were calculated in blood vs TIL. Treg were quantified as the percentage of CD25/FoxP3 double-positive cells within CD3/CD4 T-cell population in blood vs TIL. Means of CD25/FoxP3 percentages are depicted as a line (blue for HPV negative and red for HPV positive) within the plots; *P*-values are from non-parametric repeated-measures (blood, TIL) models of each cell type. ((+)=HPV positive, *N*=17 and average age is 59.4 years), ((o)=HPV−, *N*=12 and average age is 62.4 years); there was no significant difference observed for the HPV-positive vs -negative groups in blood or TIL.

We analyzed a similar number of HPV-positive and -negative head and neck patient tumors from several different anatomical locations, as shown in Table 1 and Figure 1. Both PBL and TIL were analyzed as shown in Figure 2 and the gating scheme and dot plots are shown for a representative patient. This is the gating scheme used for all the graphs throughout the Results section (Figures 3) to determine percentage of positive and mean fluorescent intensity (MFI) of proteins on T-cell subsets in both the blood and TIL.

Initially, we compared the frequency of Tregs, defined as FoxP3^+^CD25^+^CD4^+^ cells, in the blood vs tumor in SCCHN patients. The frequency of Tregs within the total CD4^+^ T-cell compartment was found to be significantly higher in TIL from 29 patients when compared with circulating lymphocytes (*P*<0.0001; Figure 3). There was no statistical difference in Treg frequency in the tumor and blood based on HPV status (*P*=0.221).

**Figure 4:**
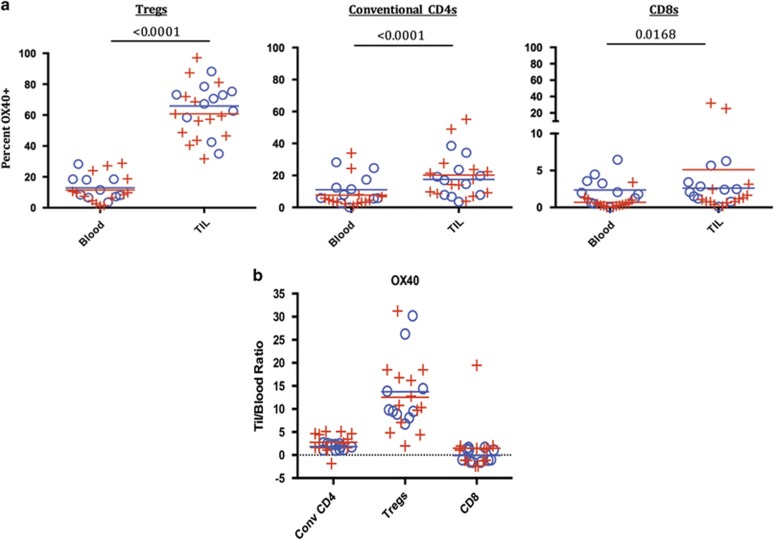
OX40 expression on Treg, CD4 and CD8 T cells in HPV-positive and -negative patients. (**a**) The percentages of OX40-positive T cells were quantified as shown in Figure 3 for Tregs, conventional CD4s and CD8s. Means for the HPV-positive and -negative groups are depicted as red and blue lines, respectively, in the plots; *P*-values are from non-parametric repeated-measures (blood, TIL) models of each cell type. ((+)=HPV positive, *N*=14), ((o)=HPV−, *N*=10); *P*-values calculated between lymphocytes isolated from HPV+ vs – in TIL and blood showed no significant differences. (**b**) MFI: Tregs, convention CD4s and CD8 T cells were gated on as individual populations and evaluated for OX40 MFI in the blood and TIL of head and neck cancer patients. No significant differences in OX40 MFI was found between HPV-positive and -negative patients (data not shown). Because of physical changes in our flow cytometer over the course of the study, the fold-change in MFI of TIL relative to blood was used to normalize the MFI data set and is shown for each subset of T cells. In a subset of patient samples with consistent cytometry settings, there was significant increases in OX40 MFI in TIL relative to blood in the Treg population (*P*=0.0039) and conventional CD4 population (*P*=0.0391); no significant difference was seen in the CD8 population (data not shown).

We next examined OX40 expression on T-cell populations from the TIL vs circulating lymphocytes in 25 subjects (HPV+ *N*=14; HPV− *N*=11). OX40 was expressed at significantly higher frequency in the Treg TIL population compared with peripheral Tregs (*P*<0.0001) (Figure 4a). We also noted that the MFI of OX40 staining was greatest on the Treg TIL population compared with all other T-cell populations analyzed in the tumor or periphery. The differences in Treg OX40 expression between the TIL and PBL were independent of HPV status or age. The frequency of OX40-expressing T cells within the conventional CD4 population was also higher in the tumor compared with PBL (*P*<0.0001). OX40 expression was low for the CD8 cell population in both the PBL and TIL. Higher expression in TIL CD8s was very small in terms of frequency as shown in Figure 4a, though consistently observed and thus was statistically significant (blood vs TIL *P*=0.0168). In addition, we found a statistical increase in MFI for OX40 expression in TIL vs blood for the Tregs and conventional CD4s but not in the CD8 T-cell population. We also measured the fold-increase in MFI for OX40 comparing TIL vs blood in each patient (Figure 4b) and found that the Tregs showed the greatest increase in OX40 MFI.

**Figure 5:**
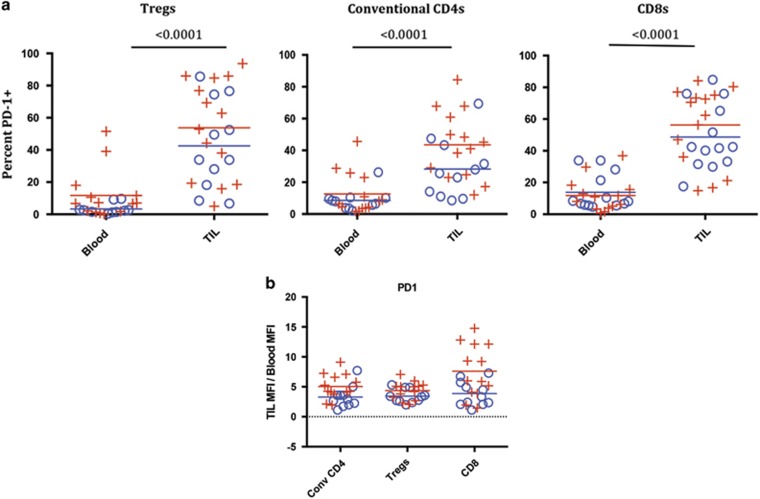
PD-1 expression on Treg, CD4 and CD8 T cells in HPV-positive and -negative patients. (**a**) The percentages of PD-1-positive T cells were quantified as shown in Figure 3 for Tregs, conventional CD4s and CD8s. Means for the HPV-positive and -negative groups are depicted as red and blue lines, respectively, in the plots; *P*-values are from non-parametric repeated-measures (blood, TIL) models of each cell type. ((+)=HPV positive, *N*=14), ((o)=HPV−, *N*=11) *P*-values for HPV+ vs − were not significant. (**b**) MFI: Tregs, convention CD4s and CD8 T cells were gated on as individual populations and evaluated for PD-1 MFI in the blood and TIL of head and neck cancer patients. Because of physical changes in our flow cytometer over the course of the study, the fold-change in MFI of TIL relative to blood was used to normalize the MFI data set and is shown for each subset of T cells. As in Figure 4, in a subset of samples with consistent cytometer settings there was significant differences in MFI in the Treg population (*P*=0.0313) and conventional CD4 population (*P*=0.0313), as well as the CD8 population (*P*=0.002) when the TIL were compared with blood (data not shown).

PD-1 expression pattern was different than that of OX40 on T-cell subsets. In general, we observed that the frequency and MFI of PD-1^+^ T cells was increased on all TIL subsets when compared with peripheral T cells (Figures 5a and b). The percentage of PD-1-expressing T cells was increased on TIL relative to the PBL T cells in Tregs (*P*<0.0001), conventional CD4 (*P*<0.0001) and CD8 T-cell populations (*P*<0.0001), and this increase was independent of HPV status (HPV+, *N*=14 and HPV−, *N*=11) or age (Figure 5a). We also found a significant increase in PD-1 MFI on all TIL subpopulations when compared with the peripheral T cells (Figure 5b).

**Figure 6:**
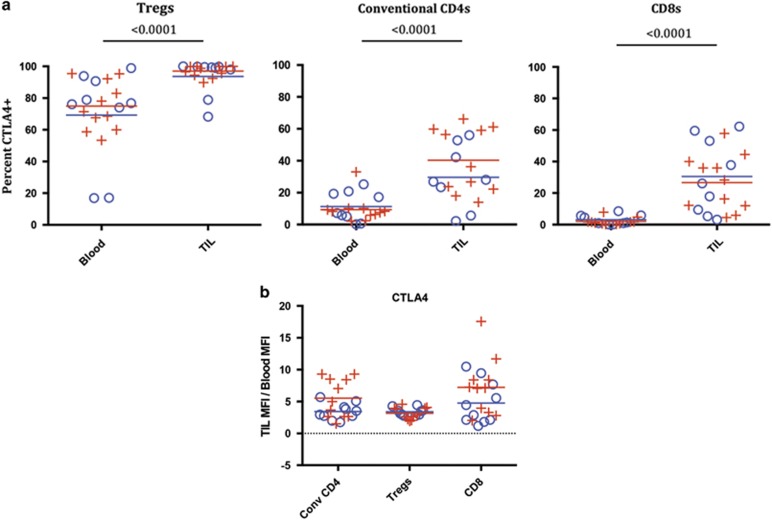
Intracellular CTLA-4 expression on Treg, CD4 and CD8 T cells in HPV-positive and -negative patients. (**a**) The percentages of CTLA-4-positive T cells were quantified as shown in Figure 3 for Tregs, conventional CD4s and CD8s. Means for the HPV-positive and -negative groups are depicted as red and blue lines, respectively, in the plots; *P*-values are from non-parametric repeated-measures (blood, TIL) models of each cell type. ((+)=HPV positive, *N*=11), ((o)=HPV−, *N*=9) *P*-values for HPV+ vs − were not significant. (**b**) MFI: Tregs, convention CD4s and CD8 T cells were gated on as individual populations and evaluated for CTLA-4 MFI in the blood and TIL of head and neck cancer patients. Mean MFI are depicted as red and blue lines in the plots for mean fold-changes. Because of physical changes in our flow cytometer over the course of the study, the fold-change in MFI of TIL relative to blood was used to normalize the MFI data set and is shown for each subset of T cells. No significant difference in fold-increase for CTLA-4 MFI was found between HPV-positive and -negative patients. In a subset of samples with consistent cytometer settings, there was significant differences in the Treg population blood vs TIL (*P*=0.0002) and in the conventional CD4 population blood vs TIL (*P*=0.00039), as well as in the CD8 population (*P*=0.0002) (data not shown).

We found that intracellular CTLA-4 expression in SCCHN patients showed similarities and differences when compared with OX40 and PD-1 expression. One major difference was the high expression level of CTLA-4 on peripheral Treg, which was not observed for OX40 or PD-1. However, the MFI of CTLA-4 on the peripheral Tregs was lower than tumor-isolated Tregs (Figure 6b), demonstrating differential expression of CTLA-4 expression within tumor Tregs. The fold-increase in TIL vs blood MFI for CTLA-4 was comparable in all T-cell subsets (Figure 6b). Similar to PD-1 expression, CTLA-4 was increased on all T-cell subsets (percentage of positive T cells as well as MFI) within the tumor compared with blood (Figure 6a). The highest level of CTLA-4 expression was found on tumor Tregs, which was similar to the OX40 expression pattern (both in percentage of positive cells and MFI). As with the other two markers, we found that there was no significant difference in CTLA-4 expression with regards to HPV status (HPV+ *N*=11; HPV− *N*=9).

**Figure 7:**
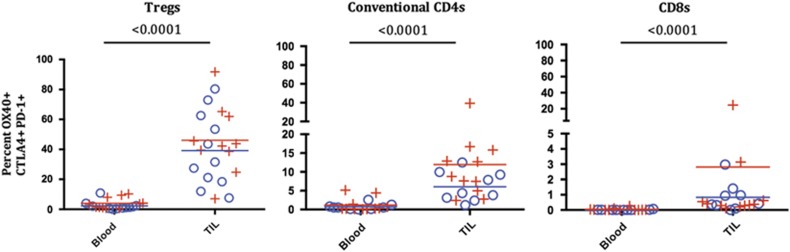
Co-expression of OX40-, CTLA-4- and PD-1-positive T cells, stratified by HPV status. The percentage of triple-positive T cells was examined in Treg, conventional CD4s and CD8s. Lymphocytes isolated from the TIL and blood were gated as in Figure 2, and for this analysis OX40^+^ cells were assessed for co-expression of PD-1 and CTLA-4. Mean values of triple-positive T cells are depicted as red and blue lines in the plots, *P*-values are from non-parametric sign test (blood, TIL) models of each cell type. ((+)=HPV positive, *N*=11), ((o)=HPV−, *N*=9).

We next examined co-expression of OX40, PD-1 and CTLA-4 on T-cell subsets by first gating on OX40^+^ cells and then selecting the CTLA-4^+^, PD-1^+^ double-positive population within the OX40^+^ gate. We then calculated the frequency of the triple-positive cells within each of the T-cell subsets. The triple-positive cells were more common within CD4 T-cell populations and relatively rare within the CD8 T-cell population. The highest levels of PD-1, CTLA-4 and OX40 triple-positive cell was found in the Treg population of TIL samples compared with the circulating lymphocytes or other TIL populations (*P*<0.0001) (Figure 7). In TIL, OX40, CTLA-4 and PD-1 triple-positive cells were found on average in 0.7% of the CD8 population, 7.5% of the conventional CD4 population and 44.8% of the Treg population (Figure 7). There were no statistical differences found in the frequency of triple-positive cells between HPV+ and HPV− patients.

**Figure 8:**
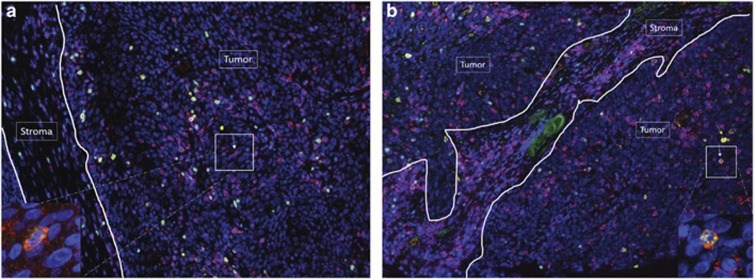
OX40 is preferentially expressed in the tumor compartment of head and neck squamous cell carcinoma. Multiplex fluorescent immunohistochemistry was performed on two SCCHN tumor specimens (**a**, **b**). Five markers were evaluated, including PD-1 (red), OX40 (yellow), FoxP3 (green), CD3 (magenta) and DAPI (blue). Arrows are pointing to cells with the following phenotypes. Yellow arrow: OX40^+^CD3^+^FoxP3^+^ cell; red arrow: OX40^+^CD3^+^FoxP3^−^ cell; magenta arrow: OX40^−^CD3^+^FoxP3^+^ cell; white arrow: OX40^+^CD3^+^PD-1^+^ cell. The insets points to cells co-expressing OX40 (yellow) and PD-1 (red).

Finally, we examined the expression of OX40 and PD-1 within the tissue of SCCHN patients by immunohistochemistry (Figure 8). We performed this analysis to understand the anatomical location of OX40- and PD-1-positive lymphocytes in relation to cancer cells and stroma within the TME. Immunohistochemical samples from two representative patients are shown, and OX40^+^ T cells were found mainly in the tumor, while PD-1-positive T cells were found both in the stroma and tumor. Similar to the flow cytometric analysis, we found that the brightest OX40 expression also co-expressed FoxP3^+^ (Treg). Also, as observed by flow cytometry, the majority of PD-1-positive T cells did not co-express OX40; however, there was a low percentage of T cells that did co-express OX40 and PD-1.

## Discussion

This is the first study to provide a detailed characterization of the three immunotherapy-based markers OX40, PD-1 and CTLA-4 together in SCCHN patients. We found a high level of expression for all three proteins selectively on T cells isolated from tumors, regardless of HPV status. There are currently clinical trials in SCCHN patients with agents that target all three of these proteins. Hence, the expression data within these patients provide knowledge that will help design scientifically sound trials in the future, which will hopefully lead to approval of these agents for SCCHN patients. We found in this study that the highest level of OX40 protein expression was in CD4^+^Foxp3^+^CD25^+^ Treg population isolated from tumors (percentage of cells and MFI), and this observation was similar in both HPV-positive and -negative patients. In contrast, the percentage of PD-1 expression was increased on all T-cell subsets (Treg, conventional CD4s and CD8s) within the tumor compared with blood, again independent of HPV status. Finally, CTLA-4 expression was increased on all T-cell subsets found within the tumor, similar to PD-1 expression, but was expressed the highest on the Tregs as was observed with OX40 expression.

Two recent reports have investigated checkpoint inhibitor expression on T cells within the TIL of SCCHN patients.^21, 22^ In particular, these manuscripts show an upregulation of PD-1 and CTLA-4 within the TME on T cells compared with PBL, although neither of these articles investigated OX40 expression. It should be noted that CTLA-4 blockade combined with PD-1 blockade showed clinical efficacy compared with Investigator's Choice in the Checkmate-141 trial (http://www.businesswire.com/news/home/20160128005239/en/CheckMate—141-Pivotal-Phase-3-Opdivo-nivolumab). Some of our findings support those of Jie *et al.*,^21^ who demonstrate that the frequency of checkpoint receptors CTLA-4^+^, TIM-3^+^ and PD-1^+^ was significantly increased in the TIL Tregs compared with PBL Tregs.^21^ In addition, the investigators found that immune-suppressive molecules, such as CD39^+^ and LAP^+^ (transforming growth factor-β-binding protein) cells, were also overexpressed in TIL Tregs when compared with PBL Tregs. The observation that CD39 and CTLA-4 are co-expressed in the majority of intratumoral Treg suggests that these two molecules may be key regulators of functional FoxP3^+^ Tregs in the tumor. Tregs have immune-suppressive properties, and although a number of studies suggest that intratumoral FoxP3^+^ Treg may be associated with poor prognosis,^23^ little is definitively known about the mechanisms of Treg activation in human tumors.

It has been proposed that CTLA-4, OX40 and PD-1 all have important and potentially distinct roles in Treg biology.^24^ It may be that the function of these proteins on Tregs could also differ depending on the immune environment (tumor vs periphery). There is evidence from human clinical trials that CTLA-4 blockade leads to peripheral Treg destabilization as a high frequency of patients experience gut toxicity and other autoimmune side effects.^25^ Little gut toxicity has been observed with the PD-1 blockade trials and this activity was not observed in patients treated with an OX40 agonist.^19^ Hence, although CTLA-4 blockade may have a role in peripheral tolerance potentially through Treg destabilization, all three immune-stimulatory strategies may have a role to destabilize Treg function or deplete Tregs within the tumor. As far as OX40 agonists are concerned, they have been shown to enhance proliferation in conventional CD4 and CD8 T cells,^19^ and perhaps the proliferation and activity of the non-Treg after anti-OX40 administration overcomes Treg inhibition, leading to tumor destruction. Recent reports have also shown that OX40 agonists can inhibit both Treg function and the generation of transforming growth factor-β-inducible Treg.^19, 26^ However, there have also been two reports that OX40 agonists can enhance Treg proliferation.^27, 28^ The percentage of Tregs and/or their expression of the proteins studied herein may change following administration of anti-OX40, anti-PD-1 or anti-CTLA-4 to cancer patients, potentially leading to clues for the timing of co-therapies.

Therapies that target these three proteins can also enhance effector T-cell function, and as alluded to above, it may be that these induced increases in T-cell effector function and proliferation can overcome Treg-mediated suppression. We tend to favor a model where enhancing immunity through these pathways most likely has effects on both T-cell effector function and Treg-mediated inhibition, although likely through distinct intracellular mechanisms. Preclinical models and clinical trials have shown that combinations of agents targeting these pathways show additive and synergistic therapeutic activity.^29, 30^ Hence, understanding the pattern of protein expression in humans shown in this study may lead to novel ways to manipulate their function *in vivo* when these agents are combined in future trials.

Evidence is accumulating that an inflammatory T-cell infiltrate and preexisting tumor-specific T-cell response are a prerequisite for a favorable outcome of immunotherapy as well as to conventional therapeutic interventions.^31, 32^ Because of the increased expression of OX40, as well as CTLA-4 and PD-1 in the TIL of SCCHN patients shown in this investigation, we hypothesize that modulating these pathways will be therapeutically beneficial in both the HPV-positive and -negative populations. We aim to test this hypothesis in a series of clinical trials that are currently in the planning stages. In these trials, we will try to induce an inflammatory T-cell response through agents that target the immune-modulatory proteins described in this manuscript, which will likely increase clinical responses in patients when combined with conventional treatment and using immunotherapy alone. Ultimately, we are pursuing a clinical strategy directed at promoting T-cell effector function and memory prior to conventional treatment, with the potential to induce anti-SCCHN immunity, lift suppressive conditions in the microenvironment and ultimately improve outcomes for patients with SCCHN.

## Methods

### Patients and specimens

Peripheral autologous blood and samples of tumor were obtained from 29 patients undergoing surgery for SCCHN. The same surgeon (RBB) at Providence Portland Medical Center treated all subjects enrolled in this study, which was performed in collaboration with investigators in the Earle A. Chiles Research Institute at the Providence Cancer Center. All subjects signed written informed consent approved by the Providence Portland Medical Center Institutional Review Board (IRB protocol no. 06-108A). After the flow cytometric analyses, patients were stratified by HPV status for the purposes of comparison. As expected, this resulted in unequal distribution of site among the comparison groups, with oropharyngeal subsites being predominant in the HPV-positive group (*N*=17) and the oral cavity and larynx most prevalent in the HPV-negative group (*N*=12) (Table 1). In a two-sample *t*-test, there was no significant difference in age between the two groups (*P*=60; HPV+, mean age=59.4 years; HPV− mean age=62.4 years) (Figure 1).

### Specimen collection

A sample of tissue was obtained from the resection or biopsy specimen of the primary tumor and immediately sent to the laboratory with 30 cc of autologous blood, which was drawn into heparinized tubes immediately before or during the surgical procedure. Tumor was minced and subjected to enzymatic digestion. Blood and tumor samples were separated with Ficoll gradient to enrich for lymphocytes. Typically, 1 × 10^7^ lymphocytes per gram of tissue was collected. Samples were frozen at −140 °C (liquid nitrogen) and later thawed in batches for analysis. Cells were then labeled with a live/dead stain and subsequently with fluorescently conjugated Abs and analyzed by flow cytometry (Figure 2). An average viability of 77.4% was observed for TIL and of 87.9% for PB mononulcear cells. Gating for OX40, CTLA-4 and PD-1 was established using fluorescent minus one (FMO) controls. These control samples were stained on TIL to accurately establish gating boundaries. An experiment was conducted to compare isotype controls vs FMO controls and it was found that FMOs provided accurate gating boundaries (data not shown).

### Ab and reagents

Sample digest was completed in a 50-ml conical tube with a magnetic stir bar at room temperature for 1 h with Collagenase at 1 mg ml^−1^ (Sigma, St Louis, MO, USA, C-5138), halyuronidase at 0.5 mg ml^−1^ (Sigma, H-6254) in RPMI (Life Technologies, Carlsbad, CA, USA, 11875-093) with 0.3% human albumin (MP Biomedicals, Santa Ana, CA, USA, 823051) and 30 units ml^−1^ DNASE (Roche, Indianapolis, IN, USA, 04536282001). Following digest, samples were filtered through a 70-μm filter. Samples were then diluted 1:2 with RPMI and layered onto Ficoll (GE, Pittsburgh, PA, USA, 17-1440-02) to enrich for lymphocytes through a centrifugation step. The cells were washed two times with Dulbecco's phosphate-buffered saline. Samples were then frozen using 10% dimethyl sulfoxide (Life Technologies, (D12345) in fetal bovine serum. Samples were thawed, and prior to surface staining cells, Fc blocking was performed using eBioscience reagents (14-9161-73). The following Abs were used for flow cytometric analysis: LiveDead e506 (eBioscience, San Diego, CA, USA, 65-0866), CD3 APC-H7 (BD, San Jose, CA, USA, 560176), CD14 AF-700 (Biolegend, San Diego, CA, USA, 325614), CD4 BV785 (Biolegend, 100551), CD8 BV711 (Biolegend, 301043), CD25 BV650 (Biolegend, 302633), FoxP3 e450 (eBioscience, 236A/E7), OX40 PE (ebioscience, 12-1247-42) PD-1 FITC (BD, 557860), and CTLA-4 APC (BD, 555855). FoxP3 staining was completed using the eBioscience FoxP3 Fix/Perm Kit (00-5523-00).

### Statistical analysis

A *t*-test was used to assess age differences in HPV groups. To assess whether T cells isolated from blood were different from TIL in the flow cytometric experiments, a repeated-measures mixed-effects model was used for each outcome. Subjects who were missing only one of either blood or TIL measures were still included in the model. Mean TIL and T cells from blood were compared while controlling for age and presence of HPV. Within-person correlation was accounted for by treating each person as a random effect. Unstructured variance–covariance was specified to incorporate separate variance estimates of T cells in the blood and TIL measurements. A restricted maximum likelihood was used to solve the equations. Due extreme outliers, non-parametric analysis was used by analyzing the global ranks of all outcomes. Fifteen outcomes were tested. With 15 tests using 0.05 level of significance, 1 significant spurious finding is expected if in fact there are no differences in the population. We used the SAS 9.3 (SAS Institute Inc., Cary, NC, USA) statistical software package for the analyses.

## Figures and Tables

**Table 1:** Location of surgery

*Location of surgery*	*HPV+ (n)*	*HPV− (n)*
Base of tongue	7	1
Tonsil	7	
Oropharynx	1	
Nasal	1	
Oral tongue	1	3
Mandibular gingiva		3
Maxillary sinus		1
Larynx		1
Floor of mouth		3

Abbreviation: HPV, human papilloma virus.
